# Trends in Atrial Fibrillation‐Related Mortality Among Older Adults With Obstructive Sleep Apnea in the United States, 1999–2020

**DOI:** 10.1002/clc.70178

**Published:** 2025-07-15

**Authors:** Ibrahim Nagmeldin Hassan, Mohamed Ibrahim, Siddig Yaqub, Muhsin Ibrahim, Haythem Abdalla, Ghada Aljaili, Wafa Osman, Nagmeldin Abuassa, Hamza Ashraf, Maryam Shoukat

**Affiliations:** ^1^ University of Khartoum Faculty of Medicine Khartoum Sudan; ^2^ Al‐Neelain University Faculty of Medicine Khartoum Sudan; ^3^ Karary University, Faculty of Medicine Khartoum Sudan; ^4^ Sudan University of Science and Technology, Faculty of Medicine Khartoum Sudan; ^5^ University of Bahri, Faculty of Medicine Khartoum Sudan; ^6^ Omdurman Islamic University, Faculty of Medicine Khartoum Sudan; ^7^ Allama Iqbal Medical College Lahore Pakistan; ^8^ Gujranwala Medical College Gujranwala Pakistan

**Keywords:** age‐adjusted mortality rate, atrial fibrillation, CDC WONDER, mortality trends, obstructive sleep apnea, older adults

## Abstract

**Background:**

Atrial fibrillation (AF) and obstructive sleep apnea (OSA) frequently coexist and synergistically increase cardiovascular risk. While their pathophysiologic interplay is well established, national data on mortality trends involving both conditions are scarce.

**Methods:**

We analyzed mortality data from the CDC WONDER platform (1999–2020), including adults aged ≥ 25 years with AF (ICD‐10 I48.x) listed as the underlying cause of death and OSA (G47.33) as a contributing condition. Age‐adjusted mortality rates (AAMRs) and average annual percent changes (AAPCs) were calculated using Joinpoint regression, stratified by sex, race/ethnicity, urbanization, region, and age.

**Results:**

A total of 32,142 AF‐related deaths with OSA were identified. The overall AAMR was 0.60 per 100 000, increasing significantly over time (AAPC: 16.69%, 95% CI: 15.62–17.77). Mortality rose across all demographic groups, with the steepest increases among adults ≥ 85 years (AAPC: 19.40%), females (AAPC: 17.77%), rural residents (AAPC: 17.51%), and White individuals (AAPC: 16.95%). Regionally, the Midwest (AAMR: 0.79) and West (0.72) had the highest rates. State‐level variation ranged from 1.90 (Oregon) to 0.19 (Mississippi). Despite lower absolute AAMRs among Hispanic and Asian populations, significant upward trends were observed. OSA appears frequently underdiagnosed or untreated in high‐risk groups, potentially exacerbating AF mortality.

**Conclusions:**

AF‐related mortality involving OSA has risen sharply over the past two decades, outpacing many other cardiovascular trends. These findings underscore the urgent need for integrated AF‐OSA screening and treatment pathways, with attention to underserved and disproportionately affected populations.

**Abbreviations**
AAMR: Age‐adjusted mortality rate
AAPC: average annual percent change
AF: atrial fibrillation
APC: annual percent change
CDC: Centers for Disease Control and Prevention
CI: confidence interval
CPAP: continuous positive airway pressure
ICD‐10: International Classification of Diseases, 10th Revision
IRB: institutional review board
NCHS: National Center for Health Statistics
OSA: obstructive sleep apnea
STROBE: Strengthening the Reporting of Observational Studies in Epidemiology
US: United States
WONDER: Wide‐ranging Online Data for Epidemiologic Research

## 1 Introduction

Atrial fibrillation (AF) is the most common sustained cardiac arrhythmia globally and a leading cause of morbidity and mortality in aging populations. In the United States, AF‐related deaths have increased substantially over the past two decades, particularly among younger adults and non‐white populations, reflecting both changing epidemiology and improved detection efforts [1].

Obstructive sleep apnea (OSA), a prevalent sleep‐disordered breathing condition marked by recurrent upper airway obstruction during sleep, is frequently underdiagnosed despite its growing contribution to cardiovascular morbidity [2]. Mounting evidence has implicated OSA as a key modifiable risk factor for the development and progression of AF [3]. Mechanistic studies suggest that OSA‐induced intermittent hypoxia, increased sympathetic tone, negative intrathoracic pressure, and oxidative stress collectively promote atrial remodeling and autonomic dysregulation, fostering an arrhythmogenic substrate [4].

This relationship is clinically significant. Individuals with OSA have an ~88% higher risk of developing AF compared to those without OSA [5, 6]. Furthermore, OSA is associated with higher rates of AF recurrence following both cardioversion and catheter ablation procedures [7]. Treatment with continuous positive airway pressure (CPAP) has been shown to reduce AF recurrence and improve cardiovascular outcomes, highlighting the importance of recognizing and addressing OSA in AF management strategies [8].

Despite the strong biological and clinical connections between AF and OSA, few studies have explored population‐level trends in AF‐related mortality among individuals with OSA. Such analyses are critical for informing targeted screening, prevention, and health policy strategies. To address this gap, we conducted a nationwide mortality trend analysis using CDC WONDER data from 1999 to 2020. Our objective was to examine temporal patterns and demographic disparities in AF‐related deaths where OSA was listed as a contributing cause [8].

## 2 Methods

### 2.1 Study Design

We conducted a cross‐sectional time‐trend analysis using mortality data from the Centers for Disease Control and Prevention (CDC) Wide‐ranging Online Data for Epidemiologic Research (WONDER) platform, a publicly accessible system that compiles death certificate data for all U.S. residents [9]. The study period spanned from 1999 to 2020, during which we examined trends in atrial fibrillation (AF)‐related mortality among adults aged 25 years and older in whom obstructive sleep apnea (OSA) was also listed as a contributing cause.

Underlying causes of death were identified using the International Classification of Diseases, Tenth Revision (ICD‐10) code I48.x, encompassing all forms of atrial fibrillation and flutter [10]. OSA was captured using the ICD‐10 code G47.33, which includes both adult and pediatric forms of the condition. We included all decedents for whom AF (I48.x) was recorded as the underlying cause of death and OSA (G47.33) appeared anywhere among the contributing causes. Cases involving central sleep apnea (G47.31) or other arrhythmias (e.g., I47, I49) were excluded to maintain diagnostic specificity (Supporting Information Table S1).

Because this study utilized publicly available and fully deidentified data, institutional review board (IRB) approval was not required. The study followed the STROBE guidelines for cross‐sectional observational studies (Supporting Information Table S2) [11].

### 2.2 Data Extraction

We extracted mortality data for the entire U.S. population aged 25 years and older, stratified by sex, ten‐year age group, race/ethnicity, U.S. Census region (Northeast, Midwest, South, West), and urbanization status (metropolitan vs. non‐metropolitan counties). Data were downloaded in April 2025.

Race and ethnicity classifications were based on death certificate records provided by funeral directors, typically informed by next of kin. Urban‐rural status was defined using the 2013 National Center for Health Statistics Urban–Rural Classification Scheme for Counties, which designates counties with populations ≥ 50 000 as metropolitan (urban) and those with fewer as non‐metropolitan (rural) [12].

In accordance with CDC WONDER data‐use standards, annual records with fewer than 10 deaths were excluded due to statistical unreliability. Consequently, temporal trend analyses for certain subgroups—such as American Indian or Alaska Native individuals and the 25–34‐year age group—were limited to years where case counts met reporting thresholds. Nonetheless, crude mortality rates were calculated for all age groups across the full study period, including suppressed groups, to provide a comprehensive overview of disease burden.

### 2.3 Statistical Analysis

We obtained annual death counts and population estimates directly from CDC WONDER. Age‐adjusted mortality rates (AAMRs) were computed using the direct method, standardized to the 2000 U.S. standard population, and expressed per 100 000 population. Crude mortality rates were used for age‐specific descriptive comparisons. Confidence intervals (CIs) for AAMRs were calculated assuming a Poisson distribution.

Temporal trends in mortality were analyzed using Joinpoint regression, which applies segmented log‐linear models to detect changes in trend over time. We calculated annual percentage change (APC) for each segment and average annual percentage change (AAPC) across the entire period, along with 95% CIs. Significant inflection points (“joinpoints”) were identified using Monte Carlo permutation testing, with statistical significance defined as *p* < 0.05.

Trend modeling was performed using version 5.0.2 of the Joinpoint Regression Program, developed by the National Cancer Institute [13]. The algorithm begins with a single‐line model and sequentially tests whether additional joinpoints significantly improve model fit. The optimal number of segments was determined independently for each subgroup, allowing for varying temporal structures across demographic and geographic categories. APCs and CIs were computed for each interval [14].

Comparative subgroup analyses were conducted to evaluate differences in AAMRs and AAPCs by sex, age group, race/ethnicity, region, and urbanization status. Trend parallelism testing was applied to assess whether temporal patterns differed significantly across strata. All analyses and visualizations were conducted using Python (v3.10), with the pandas, matplotlib, and statsmodels libraries.

## 3 Results

### 3.1 Overall Trends

From 1999 to 2020, 32,142 deaths in the United States involved both atrial fibrillation (AF) and obstructive sleep apnea (OSA). The overall age‐adjusted mortality rate (AAMR) was 0.60 per 100 000 population (95% CI: 0.38–0.83), reflecting a growing burden (Table 1).

**Table 1:** Demographic characteristics of decedents with atrial fibrillation as the underlying cause and obstructive sleep apnea as a contributing cause of death, United States, 1999–2020.

Characteristics	Deaths (%)	AAMR (95% CI) per 100 000
**Entire Cohort**	32,139 (100%)	0.60 (95% CI: 0.38 to 0.83)
**Gender**		
Female	10,876 (33.8%)	0.36 (95% CI: 0.24 to 0.49)
Male	21,266 (66.2%)	0.93 (95% CI: 0.61 to 1.26)
**Census Region**		
Northeast	4725 (14.7%)	0.47 (95% CI: 0.31 to 0.63)
Midwest	9186 (28.6%)	0.79 (95% CI: 0.50 to 1.08)
South	9887 (30.8%)	0.50 (95% CI: 0.33 to 0.67)
West	8344 (26.0%)	0.72 (95% CI: 0.46 to 0.98)
**Race/Ethnicity**		
NH American Indian or Alaska Native	180 (0.6%)	0.52 (95% CI: 0.44 to 0.60)
NH Asian or Pacific Islander	353 (1.1%)	0.19 (95% CI: 0.17 to 0.21)
NH Black or African American	2333 (7.3%)	0.49 (95% CI: 0.47 to 0.51)
NH White	29,276 (91.1%)	0.69 (95% CI: 0.68 to 0.69)
Hispanic or Latino	975 (3.0%)	0.27 (95% CI: 0.25 to 0.29)
**Urbanization**		
Metropolitan (Urban)	25,452 (79.2%)	0.58 (95% CI: 0.36 to 0.79)
Nonmetropolitan (Rural)	6690 (20.8%)	0.73 (95% CI: 0.44 to 1.03)
**Ten‐Year Age Groups** ^a^		
25–34 years	68 (0.2%)	0.01 (95% CI: 0.01 to 0.01)
35–44 years	294 (0.9%)	0.03 (95% CI: 0.03 to 0.04)
45–54 years	1447 (4.5%)	0.16 (95% CI: 0.15 to 0.16)
55–64 years	4632 (14.4%)	0.60 (95% CI: 0.59 to 0.62)
65–74 years	9212 (28.7%)	1.80 (95% CI: 1.77 to 1.84)
75–84 years	10,560 (32.9%)	3.54 (95% CI: 3.47 to 3.61)
85+ years	5929 (18.4%)	4.96 (95% CI: 4.83 to 5.09)

abbreviation: AAMR = age adjusted mortality rate, NH = non Hispanic.

a Crude mortality rate (CR) is used for analysis instead of age adjusted mortality rates (AAMR) for age groups.

**Figure 1:**
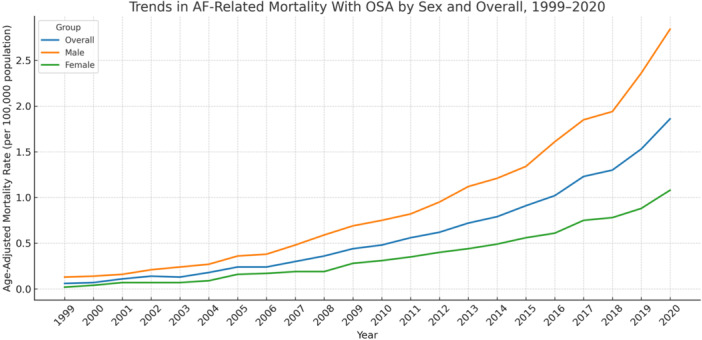
Trends in atrial fibrillation‐related mortality with obstructive sleep apnea stratified by sex and overall, United States, 1999–2020.

Joinpoint regression showed a significant and sustained increase in AF‐related mortality associated with OSA. The average annual percent change (AAPC) was 16.69% (95% CI: 15.62 to 17.77; *p* < 0.0001). Three trend segments were identified: an initial sharp rise from 1999 to 2003 (APC: 25.10%; 95% CI: 7.07 to 46.17; *p* = 0.0195), followed by steady increases from 2004 to 2012 (APC: 16.68%; 95% CI: 14.68 to 18.73; *p* < 0.0001) and from 2013 to 2020 (APC: 14.27%; 95% CI: 12.81 to 15.75; *p* < 0.0001) (Table 2; Figure 1).

**Table 2:** Annual percentage changes (APCs) and average annual percentage changes (AAPCs) in atrial fibrillation–related mortality among older adults with obstructive sleep apnea, United States, 1999–2020.

Characteristics	Trend segment	Year interval	APC (95% CI)	AAPC (95% CI)	*p* value
**Entire Cohort**				16.69 (15.62 to 17.77)	< 0.0001
	1	1999–2003	25.10 (7.07 to 46.17)		0.0195
	2	2004–2012	16.68 (14.68 to 18.73)		< 0.0001
	3	2013–2020	14.27 (12.81 to 15.75)		< 0.0001
**Gender**					
Female				17.77 (15.79 to 19.79)	< 0.0001
	1	1999–2001	87.08 (14.64 to 205.31)		0.0391
	2	2002–2004	13.39 (–54.89 to 185.04)		0.3333
	3	2005–2020	13.72 (12.85 to 14.59)		< 0.0001
Male				15.74 (15.05 to 16.42)	< 0.0001
	1	1999–2001	10.94 (–10.79 to 37.96)		0.1042
	2	2002–2009	18.99 (16.92 to 21.10)		< 0.0001
	3	2010–2020	13.90 (13.00 to 14.81)		< 0.0001
**Census Region**					
Northeast				14.84 (13.80 to 15.88)	< 0.0001
	1	1999–2004	13.62 (4.16 to 23.94)		0.0151
	2	2005–2012	16.24 (9.76 to 23.11)		0.0007
	3	2013–2020	14.18 (11.25 to 17.19)		< 0.0001
Midwest				17.82 (16.54 to 19.11)	< 0.0001
	1	1999–2004	25.79 (12.19 to 41.03)		0.0051
	2	2005–2013	17.74 (14.29 to 21.30)		< 0.0001
	3	2014–2020	15.26 (13.99 to 16.55)		< 0.0001
South				16.26 (14.68 to 17.86)	< 0.0001
	1	1999–2004	29.57 (15.41 to 45.47)		0.0034
	2	2005–2012	14.46 (11.56 to 17.44)		< 0.0001
	3	2013–2020	13.06 (10.63 to 15.54)		< 0.0001
West				16.71 (15.73 to 17.70)	
	1	1999–2004	19.97 (8.35 to 32.83)		0.0077
	2	2005–2013	14.15 (12.97 to 15.35)		< 0.0001
	3	2014–2020	14.34 (11.86 to 16.87)		< 0.0001
**Race/Ethnicity**					
NH American Indian or Alaska Native				4.12 (–16.11 to 29.22)	0.2539
	1	2017–2020	4.12 (–16.11 to 29.22)		0.2539
NH Asian or Pacific Islander				18.27 (7.99 to 29.52)	0.0069
	1	2015–2020	18.27 (7.99 to 29.52)		0.0069
NH Black or African American				13.42 (12.21 to 14.64)	< 0.0001
	1	2003–2008	17.82 (10.15 to 26.02)		0.0025
	2	2009–2020	11.71 (9.74 to 13.70)		< 0.0001
NH White				16.95 (15.76 to 18.15)	< 0.0001
	1	1999–2008	22.29 (18.12 to 26.61)		< 0.0001
	2	2009–2020	13.87 (13.27 to 14.47)		< 0.0001
Hispanic or Latino				13.28 (10.51 to 16.13)	< 0.0001
	1	2008–2010	13.39 (7.03 to 20.13)		0.0230
	2	2011–2020	15.76 (11.39 to 20.29)		< 0.0001
**Urbanization**					
Metropolitan (Urban)				16.88 (15.57 to 18.21)	< 0.0001
	1	1999–2004	27.09 (17.29 to 37.70)		0.0012
	2		2005–2013	15.29 (13.70 to 16.89)	< 0.0001
	3	2014–2020	14.11 (11.63 to 16.65)		< 0.0001
Nonmetropolitan (Rural)				17.51 (16.57 to 18.46)	< 0.0001
	1	1999–2004	17.78 (7.12 to 29.49)		0.0087
	2	2005–2012	15.71 (11.87 to 19.68)		< 0.0001
	3	2013–2020	15.72 (13.92 to 17.54)		< 0.0001
**Ten‐Year Age Groups** ^a^, ^b^					
45–54 years				9.84 (8.63 to 11.07)	< 0.0001
	1	2002–2012	10.96 (7.35 to 14.70)		0.0001
	2	2013–2020	11.28 (7.70 to 14.98)		0.0002
55–64 years				12.25 (11.27 to 13.23)	< 0.0001
	1	1999–2008	15.52 (11.78 to 19.39)		< 0.0001
	2	2009–2020	10.19 (8.63 to 11.77)		< 0.0001
65–74 years				15.00 (13.74 to 16.28)	< 0.0001
	1	1999–2009	21.08 (18.58 to 23.64)		< 0.0001
	2	2010–2020	11.94 (10.63 to 13.27)		< 0.0001
75–84 years				18.13 (16.97 to 19.31)	< 0.0001
	1	1999–2009	22.01 (18.91 to 25.19)		< 0.0001
	2	2010–2020	14.08 (12.87 to 15.31)		< 0.0001
85+ years				19.40 (18.20 to 20.62)	< 0.0001
	1	2000–2009	21.70 (15.56 to 28.16)		< 0.0001
	2	2010–2020	18.66 (17.33 to 20.01)		< 0.0001

Abbreviation: AAMR = age adjusted mortality rate, AAPC = average annual percentage change, APC = annual percentage change, NH = non Hispanic.

^a^ Trend analysis was not performed for age groups 25–34 and 35–44 years due to data suppression and statistical unreliability related to low death counts in these cohorts.

b Crude rate is used for all age dependent analysis.

### 3.2 Trends by Gender

Between 1999 and 2020, atrial fibrillation (AF) with obstructive sleep apnea (OSA) contributed to 21 266 deaths among males and 10,876 among females. The burden was notably higher in men, with an age‐adjusted mortality rate (AAMR) of 0.93 per 100 000 (95% CI: 0.59 to 1.27), compared to 0.36 per 100 000 in women (95% CI: 0.23 to 0.50) (Table 1).

Despite this difference in absolute rates, both sexes exhibited significant and sustained increases in mortality over time. Among females, the average annual percent change (AAPC) was 17.77% (95% CI: 15.79 to 19.79; *p* < 0.0001), slightly higher than that observed in males (AAPC: 15.74%; 95% CI: 15.05 to 16.42; *p* < 0.0001).

For women, the trend began with a dramatic rise between 1999 and 2001 (APC: 87.08%; 95% CI: 14.64 to 205.31; *p* = 0.0391), followed by a nonsignificant plateau from 2002 to 2004 (APC: 13.39%; 95% CI: –54.89 to 185.04; *p* = 0.3333). From 2005 onward, mortality rose steadily through 2020 (APC: 13.72%; 95% CI: 12.85 to 14.59; *p* < 0.0001).

In men, the increase was more gradual at first, with a nonsignificant rise from 1999 to 2001 (APC: 10.94%; 95% CI: –10.79 to 37.96; *p* = 0.1042). However, rates surged between 2002 and 2009 (APC: 18.99%; 95% CI: 16.92 to 21.10; *p* < 0.0001), and continued to climb steadily through the final decade of the study (APC: 13.90%; 95% CI: 13.00 to 14.81; *p* < 0.0001) (Table 2; Figure 1).

### 3.3 Trends by Race/Ethnicity

Trends in AF‐Related Mortality With OSA by Race and Ethnicity, 1999–2020

AAMRs were highest among White individuals (0.69 per 100 000; 95% CI: 0.68 to 0.69), followed by American Indian or Alaska Native (0.52; 95% CI: 0.44 to 0.60), Black or African American (0.49; 95% CI: 0.47 to 0.51), and Asian or Pacific Islander (0.19; 95% CI: 0.17 to 0.21). Hispanic or Latino individuals had an AAMR of 0.27 (95% CI: 0.25 to 0.29) (Table 1).

**Figure 2:**
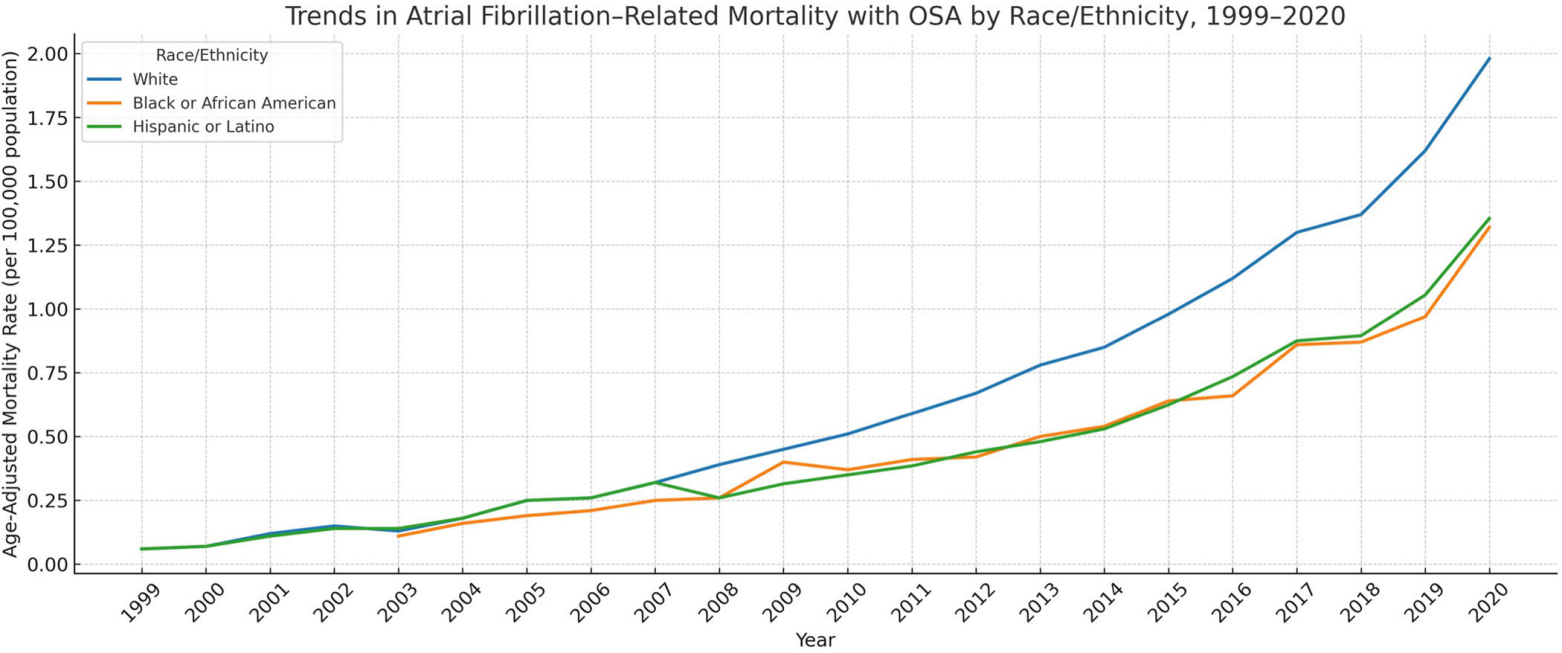
Trends in atrial fibrillation–related mortality with obstructive sleep apnea stratified by race and ethnicity, United States, 1999–2020.

White individuals had the steepest increase (AAPC: 16.95%; 95% CI: 15.76 to 18.15; *p* < 0.0001), followed by Black individuals (AAPC: 13.42%; 95% CI: 12.21 to 14.64; *p* < 0.0001), and Hispanic individuals (AAPC: 13.28%; 95% CI: 10.51 to 16.13; *p* < 0.0001). The trend for Asian individuals was also significant (AAPC: 18.27%; 95% CI: 7.99 to 29.52; *p* = 0.0069). No significant increase was observed among American Indian or Alaska Native individuals (AAPC: 4.12%; 95% CI: –16.11 to 29.22; *p* = 0.2539) (Table 2; Figure 2).

### 3.4 Trends by Urbanization

Trends in AF‐Related Mortality with OSA by Urbanization, 1999–2020

Mortality rates were higher in nonmetropolitan areas (AAMR: 0.73 per 100 000; 95% CI: 0.44 to 1.03) compared to metropolitan areas (0.58; 95% CI: 0.36 to 0.79) (Table 1).

**Figure 3:**
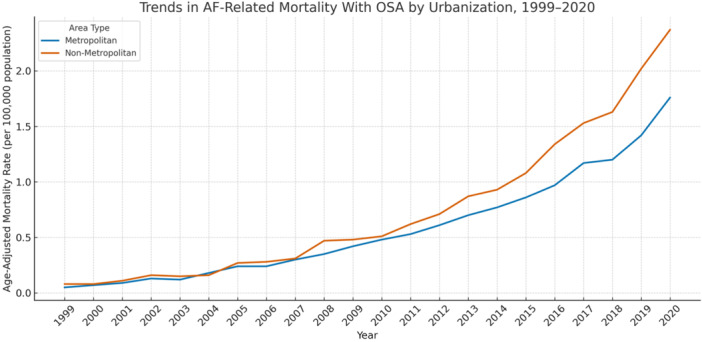
Trends in atrial fibrillation–related mortality with obstructive sleep apnea stratified by urbanization level, United States, 1999–2020.

Both settings saw significant increases over time. In metropolitan areas, the AAPC was 16.88% (*p* < 0.0001), with a sharp early rise (APC: 27.09% from 1999 to 2004), followed by steady growth. nonmetropolitan areas showed a slightly steeper trend (AAPC: 17.51%; *p* < 0.0001), with consistent increases across all three segments (Table 2; Figure 3).

### 3.5 Trends by Census Region

Trends in AF‐Related Mortality With OSA by U.S. Census Region, 1999–2020

Marked regional variation was observed in AF‐related mortality with OSA. The highest AAMR was seen in the Midwest (0.79 per 100 000; 95% CI: 0.50 to 1.08), followed by the West (0.72; 95% CI: 0.46 to 0.98), South (0.50; 95% CI: 0.33 to 0.67), and Northeast (0.47; 95% CI: 0.31 to 0.63) (Table 1). These differences may reflect variations in population comorbidity profiles, healthcare infrastructure, or regional diagnostic practices.

**Figure 4:**
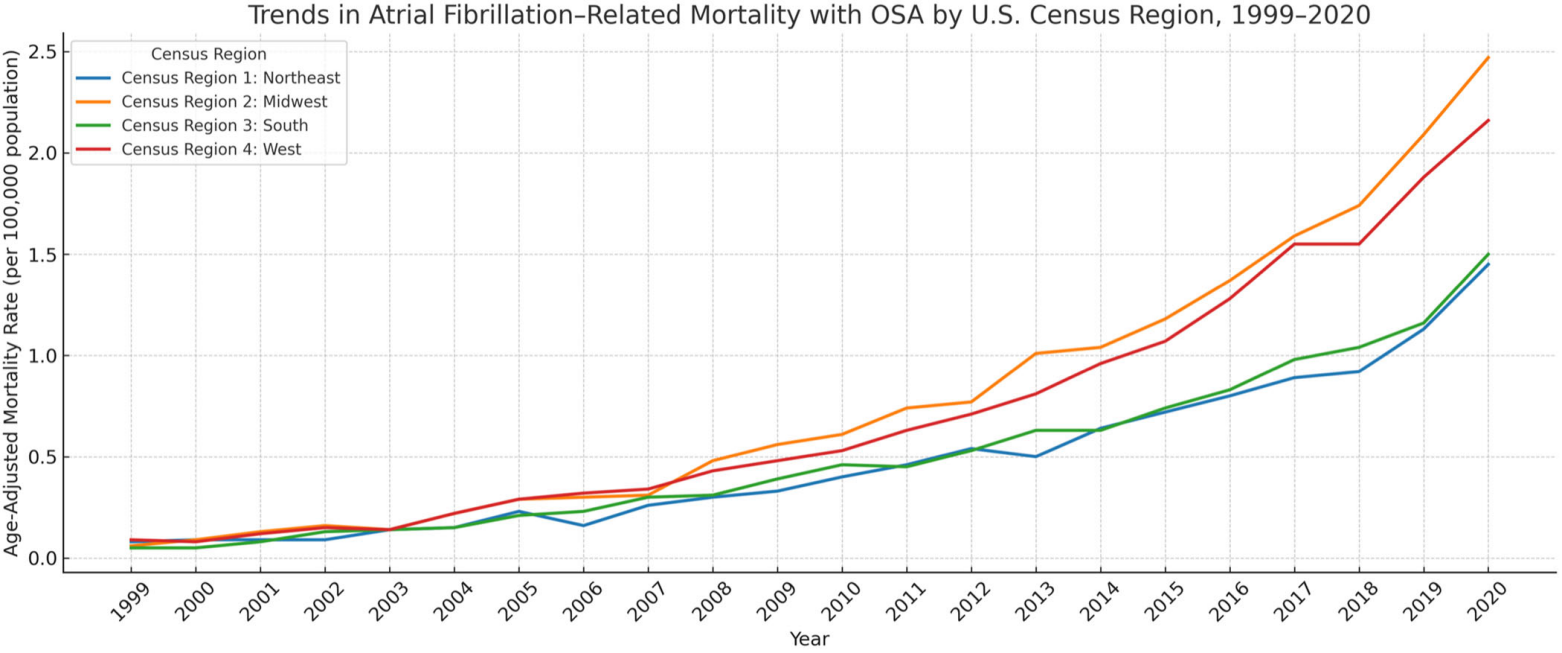
Trends in atrial fibrillation–related mortality with obstructive sleep apnea stratified by U.S. Census Region, 1999–2020.

Despite differences in absolute rates, all four regions experienced significant and sustained increases over time. The Midwest had the steepest rise (AAPC: 17.82%; 95% CI: 16.54 to 19.11; *p* < 0.0001), consistent with its high baseline burden. The West (AAPC: 16.71%; 95% CI: 15.73 to 17.70) and South (16.26%; 95% CI: 14.68 to 17.86) followed closely, suggesting parallel epidemiologic shifts. The Northeast showed the slowest increase (AAPC: 14.84%; 95% CI: 13.80 to 15.88), though still significant (Table 2; Figure 4).

### 3.6 Trends by State

State‐Level Variation in AF‐Related Mortality With OSA, 1999–2020 Pronounced variation in AF‐related mortality with OSA was observed across U.S. states. The highest AAMRs were found in Oregon (1.90 per 100 000; 95% CI: 1.79 to 2.01), Minnesota (1.71; 95% CI: 1.62 to 1.80), Montana (1.45; 95% CI: 1.27 to 1.64), Vermont (1.45; 95% CI: 1.22 to 1.68), and Wyoming (1.42; 95% CI: 1.16 to 1.68). These states—many of which are in the West and Upper Midwest—may have higher detection rates of OSA or greater awareness of AF‐OSA overlap in clinical documentation.

In contrast, the lowest AAMRs were recorded in Mississippi (0.19; 95% CI: 0.15 to 0.23), Louisiana (0.20; 95% CI: 0.17 to 0.23), and the District of Columbia (0.21; 95% CI: 0.15 to 0.29). These regional differences may reflect disparities in healthcare access, underdiagnosis of OSA, or variation in death certificate coding.

**Figure 5:**
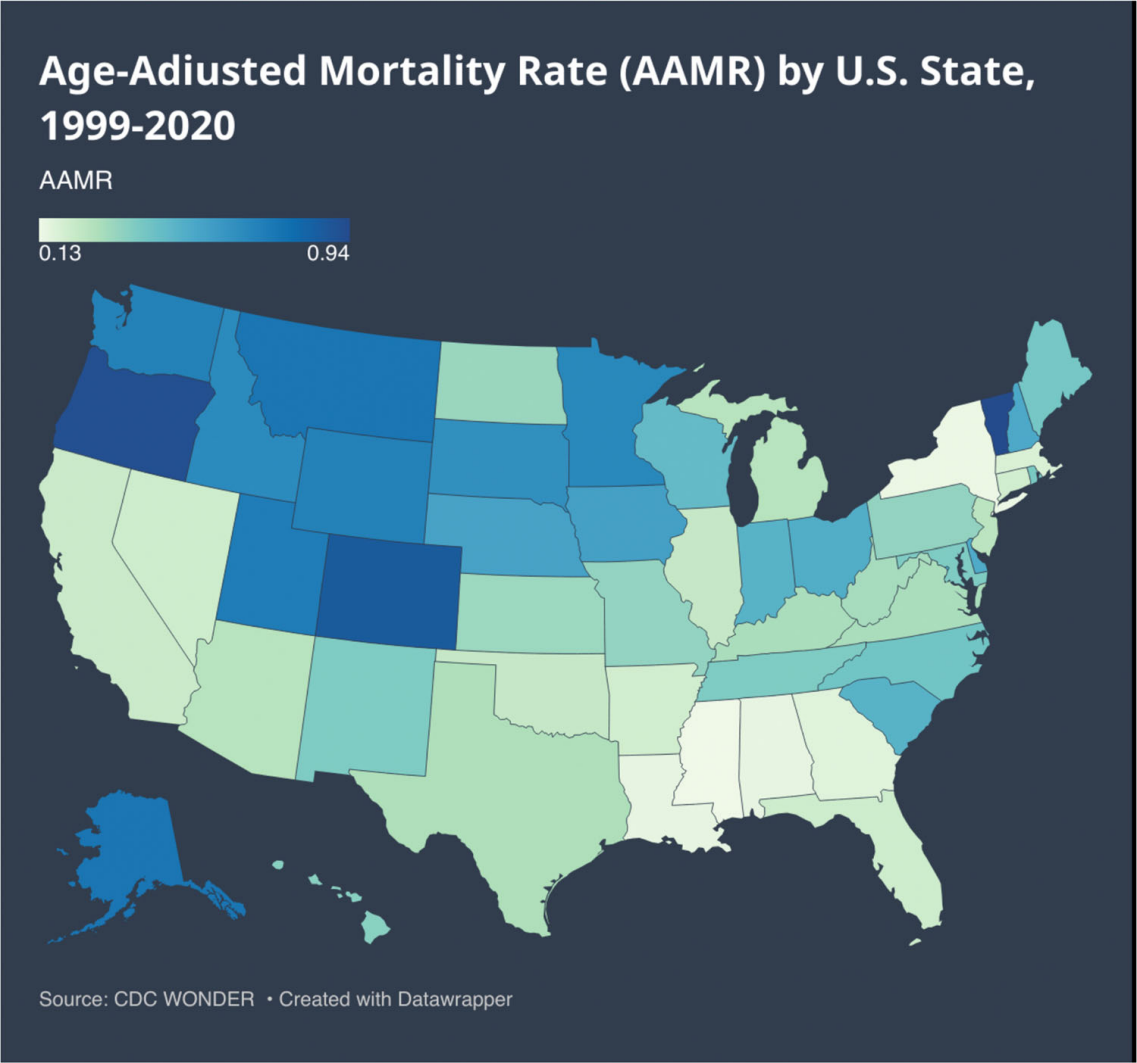
Geographic distribution of atrial fibrillation–related mortality with obstructive sleep apnea by state, United States, 1999–2020.

Overall, the wide state‐to‐state variability highlights potential inequities in disease recognition and suggests opportunities for targeted educational, diagnostic, and policy interventions (Figure 5).

### 3.7 Trends by Ten‐Year Age Group

Trends in AF‐Related Mortality With OSA by Age Group, 1999–2020

Crude mortality rates for AF with OSA rose sharply with age. The rate was lowest among individuals aged 25–34 years (0.01 per 100 000; 95% CI: 0.01 to 0.01) and increased steadily to 4.96 per 100 000 (95% CI: 4.83 to 5.09) among those aged 85 years and older (Table 1). This gradient reflects the compounding effects of age, comorbidity burden, and AF‐OSA interplay in older adults.

**Figure 6:**
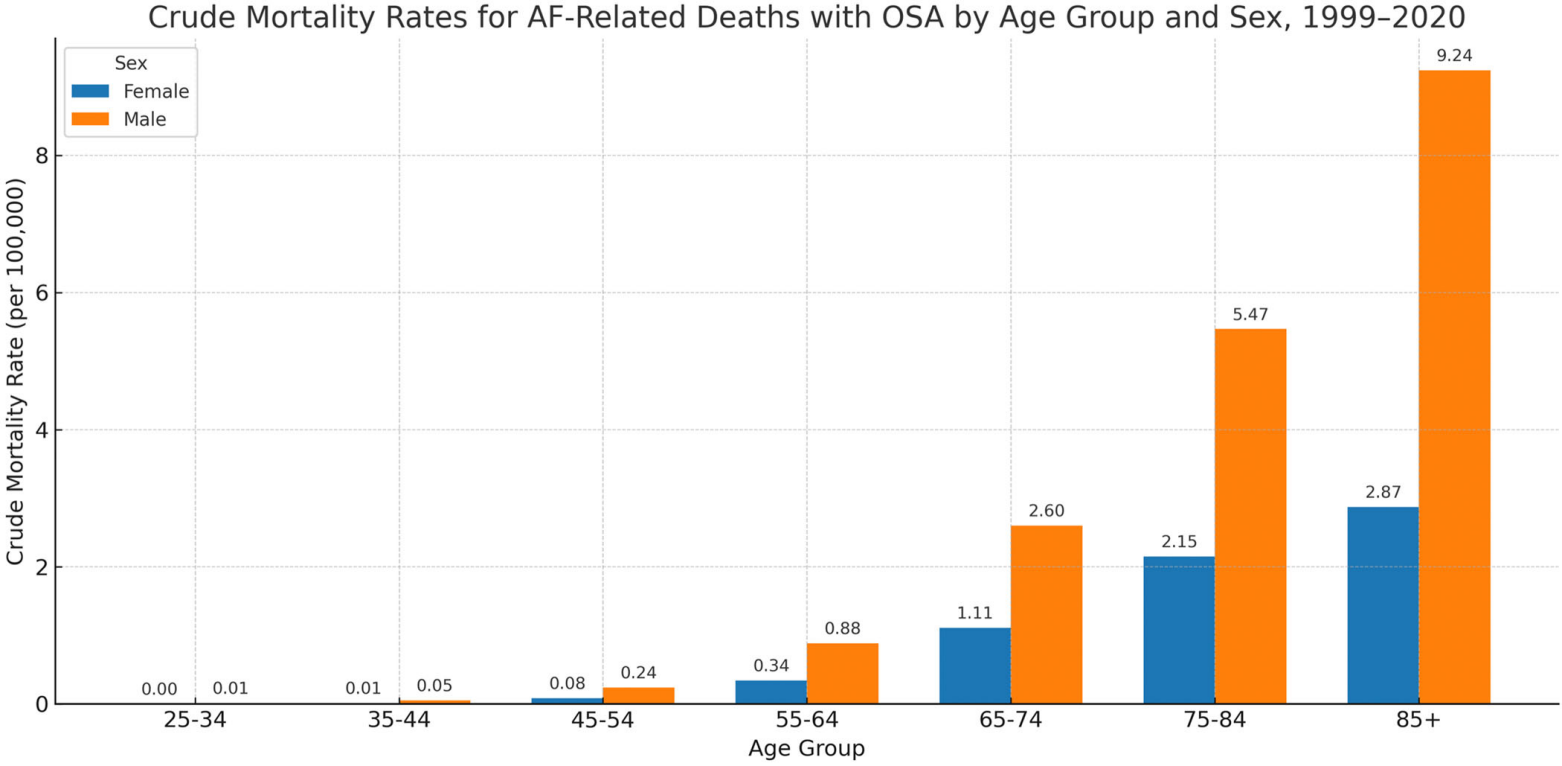
Crude mortality rates for atrial fibrillation–related mortality with obstructive sleep apnea stratified by tenten‐year‐year age groups and sex, United States, 1999–2020.

Joinpoint regression confirmed significant upward trends in all age groups ≥ 45 years. The most pronounced increase was observed in those aged ≥85, with an AAPC of 19.40% (95% CI: 18.20 to 20.62; *p* < 0.0001), followed by 18.13% in the 75–84 group (95% CI: 16.97 to 19.31; *p* < 0.0001). These findings highlight a disproportionate acceleration in mortality among the oldest cohorts—likely reflecting frailty, delayed diagnosis, or limited treatment options for OSA in late life. In the 65–74 and 55–64 groups, AAPCs were also substantial (15.00% and 12.25%, respectively; both *p* < 0.0001), suggesting a growing burden in the Medicare‐age population. Even among adults aged 45–54, mortality rose steadily (AAPC: 9.84%; 95% CI: 8.63 to 11.07; *p* < 0.0001), reinforcing that this is not solely a geriatric issue (Table 2; Figure 6).

## 4 Discussion

In this nationwide analysis spanning 1999 to 2020, we identified 32,142 deaths in which atrial fibrillation (AF) was the underlying cause and obstructive sleep apnea (OSA) was a contributing condition. The overall age‐adjusted mortality rate (AAMR) was 0.60 per 100 000, and the burden more than doubled over time, with an average annual percent change (AAPC) of 16.69%—a rate of increase that outpaces overall AF‐related mortality trends reported in prior literature [15]. This rise was consistent across demographic and geographic strata but was especially marked among individuals aged ≥ 75 years, rural residents, and those in the Midwest and West.

Our findings highlight a growing and underrecognized mortality burden related to the coexistence of AF and OSA. Although each condition independently contributes to cardiovascular morbidity and mortality, their interaction appears to potentiate risk further. Mechanistic studies have shown that OSA promotes atrial remodeling, inflammation, and autonomic dysfunction, all of which accelerate AF progression and increase complications [5, 16, 17]. Yet, until now, national‐level data quantifying this overlap in mortality trends were lacking. Our study fills that gap and underscores the need for integrated management approaches. The observed increase may be partly explained by a rising prevalence of OSA, improved survival with chronic cardiovascular disease, delayed OSA diagnosis, and suboptimal treatment adherence to CPAP therapy—all of which may exacerbate AF‐related outcomes [3, 18].

The surge in AF‐related mortality among individuals with OSA significantly outpaces national AF mortality trends. Prior studies have attributed the general rise in AF mortality to aging populations, increasing comorbidity burdens, and improved documentation on death certificates [18]. However, our findings suggest that the presence of OSA accelerates this trend, likely due to a synergistic pathophysiologic relationship. Globally, AF prevalence is expected to double by 2050, particularly in high‐income countries [19], while OSA prevalence is also on the rise due to growing rates of obesity and sedentary behavior [20]. Despite these parallel increases, few studies have evaluated how their co‐occurrence impacts population‐level mortality. Our data provide novel evidence that AF‐OSA comorbidity may drive a distinct and sharper mortality curve—one that is rising faster than that for other major cardiovascular conditions such as ischemic heart disease, stroke, and heart failure, which have plateaued or declined in recent years [21]. This disparity may reflect missed opportunities in early recognition and treatment of OSA in AF patients, highlighting a major public health concern.

The link between OSA and AF‐related mortality is biologically plausible and well‐supported. OSA induces repetitive intrathoracic pressure changes, intermittent hypoxia, and sympathetic surges, all of which promote atrial stretch, fibrosis, and electrical instability [22]. These factors hasten the progression from paroxysmal to persistent AF and increase thromboembolic risk [23].

Moreover, OSA is associated with conditions like hypertension, left atrial enlargement, and diastolic dysfunction, which compound AF‐related risks [24]. Oxidative stress and endothelial dysfunction from apneic episodes further elevate the risk of atherosclerosis and thrombosis [25]. Notably, untreated OSA may blunt the effectiveness of antiarrhythmic therapies and elevate AF recurrence after interventions like ablation or cardioversion [26]. Despite this evidence, OSA remains widely underdiagnosed in AF populations—particularly among women and minorities [27]. This under‐recognition delays the initiation of CPAP therapy, which has been shown to reduce AF recurrence and may lower cardiovascular mortality.

Despite the strong link between AF and OSA, significant care gaps remain. OSA is underdiagnosed, especially in older adults, women, and non‐white individuals [28]. Atypical presentations, limited screening, and low clinical suspicion contribute to missed diagnoses, particularly in high‐risk groups [29]. Even when diagnosed, CPAP adherence remains poor—estimated at <50% long‐term [30]. Discomfort, lack of education, and limited follow‐up are contributing factors, particularly in underserved populations. In AF management, patients with OSA are often less likely to receive anticoagulation or rhythm control therapy [31], and untreated OSA increases the risk of AF recurrence post‐ablation or cardioversion [32]. These dual deficits in recognition and treatment likely underlie the persistent rise in AF‐OSA mortality. Integrating routine OSA screening into AF pathways and expanding access to sleep diagnostics may mitigate these outcomes.

While men accounted for most AF‐OSA deaths, women experienced a slightly steeper increase in mortality over time. This discrepancy likely reflects diagnostic and biological differences. Men typically present with classic OSA symptoms, increasing the likelihood of detection [33], whereas women more often exhibit fatigue, insomnia, or mood symptoms, leading to underdiagnosis [34]. Additionally, sex‐based variations in atrial remodeling and hormone levels may influence AF progression and treatment outcomes [35]. Paradoxically, women are more symptomatic yet less likely to receive rhythm control or anticoagulation therapy [36]. Combined with delayed OSA diagnosis, these disparities may amplify long‐term mortality risks in women. Most existing AF‐OSA studies are male‐dominated, and our findings highlight the need for sex‐specific research and management approaches.

White individuals had the highest AAMRs for AF‐OSA mortality, but the annual rate of increase was also substantial among Black, Hispanic, and Asian populations. These disparities may be influenced by differences in access to care, socioeconomic status, and cultural perceptions of sleep health [37]. Minority groups are less likely to undergo polysomnography or receive OSA treatment [38], contributing to more advanced disease and poorer outcomes. Structural barriers—such as income inequality, limited insurance, and rural residence—compound these disparities [39]. Notably, some minority groups (e.g., Asian and Hispanic populations) showed lower absolute AAMRs, but this may reflect underdiagnosis rather than lower risk [40, 41]. Cultural stigma and reduced health literacy may also play a role. Culturally competent outreach and community‐based screening could improve early detection and outcomes.

Individuals in rural areas exhibited higher AAMRs and steeper mortality increases compared to those in metropolitan settings. This may reflect limited access to cardiology and sleep medicine services, fewer diagnostic facilities, and geographic isolation [42]. Rural clinicians may lack resources for complex comorbidity management, leading to delays in diagnosis and suboptimal care [43]. Regionally, the Midwest and West showed the highest mortality rates and the sharpest increases, potentially due to higher comorbidity burdens and better death certificate reporting [44, 45]. Lower rates in the South and Northeast may reflect underdiagnosis or limited integration between sleep and cardiovascular care. These patterns call for region‐specific public health efforts and infrastructure expansion.

The continued rise in AF‐OSA mortality underscores the need for integrated clinical and public health responses. Early identification and treatment of OSA in AF patients should become routine [46]. Screening tools like the STOP‐Bang questionnaire offer simple methods for identifying high‐risk individuals [47]. CPAP therapy, shown to reduce AF recurrence and improve cardiovascular outcomes, remains underused [48]. Efforts to improve CPAP adherence—including patient education and enhanced support—are critical.

On a systems level, public health campaigns should raise awareness about the AF‐OSA link, particularly among older adults, rural residents, and underserved minorities. Expanding access to diagnostic and treatment services, embedding sleep evaluations into cardiology clinics, and fostering interdisciplinary collaboration can bridge care gaps and reduce preventable deaths.

## 5 Strengths and Limitations

This study offers a comprehensive, population‐level assessment of trends in atrial fibrillation (AF)–related mortality with obstructive sleep apnea (OSA) over a 22‐year period in the United States. By leveraging the CDC WONDER database, we captured all death certificate data for U.S. residents, enabling robust estimates across a wide range of demographic and geographic subgroups. The use of Joinpoint regression further strengthened our ability to detect inflection points and quantify temporal shifts in mortality trends with statistical rigor. Importantly, our stratified analysis by sex, race/ethnicity, urbanization, region, and age group provides nuanced insights into disparities that may otherwise be obscured in aggregate data. These findings may help inform policy and clinical interventions tailored to specific high‐risk populations.

However, several limitations must be acknowledged. First, the reliance on death certificate data may introduce misclassification bias. The accuracy of coding for both AF and OSA depends on clinician documentation and local reporting practices, which can vary by institution and over time [49]. OSA, in particular, is frequently underdiagnosed and may be underreported as a contributing cause of death, potentially leading to conservative mortality estimates. Second, the dataset lacks clinical granularity. Information on comorbidities, severity of OSA, CPAP adherence, AF subtype, treatment strategies, and socioeconomic status is not available—limiting our ability to adjust for confounding factors [50]. Third, causal inferences cannot be drawn due to the observational and retrospective nature of the analysis. Lastly, subgroup trends—particularly among racial minorities or in younger age groups—should be interpreted with caution due to smaller event counts and wider confidence intervals. Nevertheless, our findings provide a valuable foundation for future research and highlight key areas for healthcare system improvement.

## 6 Conclusion

This national analysis reveals a striking and sustained rise in atrial fibrillation (AF)–related mortality among individuals with obstructive sleep apnea (OSA) in the United States from 1999 to 2020. The rapid pace of increase—particularly among older adults, women, rural residents, and minority populations—underscores the substantial and growing public health burden of this clinical overlap. Despite well‐established mechanistic links between OSA and AF progression, widespread underdiagnosis and suboptimal treatment persist, likely driving the observed mortality trends. These findings highlight an urgent need for integrated care models, routine OSA screening in AF patients, and targeted interventions aimed at improving diagnosis, treatment adherence, and equitable access to sleep and cardiovascular care services.

## Author Contributions

**Ibrahim Nagmeldin Hassan:** conceptualization, methodology, project administration, visualization, writing – original draft, writing – review and editing. **Mohamed Ibrahim:** conceptualization, formal analysis, visualization, writing – original draft, writing – review and editing. **Siddig Yaqub:** project administration, validation, writing – original draft, writing – review and editing. **Muhsin Ibrahim:** writing – original draft, writing – review and editing. **Haythem Abdalla:** project administration, investigation, data curation. **Ghada Aljaili:** writing – review and editing, project administration. **Wafa Osman:** writing – review and editing, project administration, methodology. **Nagmeldin Abuassa:** writing – original draft, writing – review and editing. **Hamza Ashraf:** writing – original draft, writing – review and editing. **Maryam Shoukat:** writing – original draft, writing – review and editing. All authors read and approved the final manuscript.

## Ethics Statement

The authors have nothing to report.

## Consent

The authors have nothing to report.

## Conflicts of Interest

The authors declare no conflicts of interest.

## Supporting information


**Supporting Information Table S1**. ICD‐10 Codes Used to Define Atrial Fibrillation and Obstructive Sleep Apnea–Related Mortality, With Inclusion and Exclusion Justifications. **Supporting Information Table S2**. Strengthening the Reporting of Observational Studies in Epidemiology (STROBE) Checklist.

## Data Availability

The data that support the findings of this study are openly available in CDC WONDER at https://wonder.cdc.gov/, reference number N/A.
