# Supplementary material for: Trends in Atrial Fibrillation‐Related Mortality Among Older Adults With Obstructive Sleep Apnea in the United States, 1999–2020
Source: Clin Cardiol. 2025 Jul 15;48(7):e70178. doi: 10.1002/clc.70178 (PMC12261039; doi:10.1002/clc.70178)
Supplement: Supplementary file 1 — Supporting Information Table S1. ICD‐10 Codes Used to Define Atrial Fibrillation and Obstructive Sleep Apnea–Related Mortality, With Inclusion and Exclusion Justifications. Supporting Information Table S2. Strengthening the Reporting of Observational Studies in Epidemiology (STROBE) Checklist. [file CLC-48-e70178-s001.docx]

Supplementary Materials

**Table S1. ICD-10 Codes Used to Define Atrial Fibrillation and Obstructive Sleep Apnea–Related Mortality, With Inclusion and Exclusion Justifications.**

| **Characteristics** | **ICD-10 Code(s)** | **Description** | **Use in Study** | **Justification** |
| --- | --- | --- | --- | --- |
| **Atrial Fibrillation (AF)** | I48.x | Atrial fibrillation and atrial flutter | Underlying cause of death | I48.x codes comprehensively capture AF-related deaths per CDC WONDER guidelines. |
| **Obstructive Sleep Apnea** | G47.33 | Obstructive sleep apnea (adult and pediatric) | Contributing cause of death | G47.33 is specific for OSA and is used widely in mortality and epidemiological data. |
| **Excluded Codes** | G47.31 | Central sleep apnea | Excluded | To isolate OSA-related burden; central sleep apnea differs in etiology and risk profile. |
|  | I47, I49 | Other supraventricular and ventricular arrhythmias | Excluded | To maintain specificity for atrial fibrillation only. |

**ICD-10 = International Classification of Diseases, 10th Revision; AF = Atrial Fibrillation; OSA = Obstructive Sleep Apnea.**

**Table S2. Strengthening the Reporting of Observational Studies in Epidemiology (STROBE) Checklist**

|  | **Item No** | **Recommendation** | **Section or**  **page number** |
| --- | --- | --- | --- |
| **Title and abstract** | 1 | (*a*) Indicate the study’s design with a commonly used term in  the title or the abstract | 1 |
|  |  | (*b*) Provide in the abstract an informative and balanced | 2 |
|  |  | summary of what was done and what was found |  |
| **Introduction** |  |  |  |
| Background/rationale | 2 | Explain the scientific background and rationale for the | 3 |
|  |  | investigation being reported |  |
| Objectives | 3 | State specific objectives, including any prespecified | 3 |
|  |  | hypotheses |  |
| **Methods** |  |  |  |
| Study design | 4 | Present key elements of study design early in the paper | 4 |
| Setting | 5 | Describe the setting, locations, and relevant dates, including | 4 |
|  |  | periods of recruitment, exposure, follow-up, and data |  |
|  |  | collection |  |
| Participants | 6 | (*a*) Give the eligibility criteria, and the sources and methods | 4 |
|  |  | of selection of participants |  |
| Variables | 7 | Clearly define all outcomes, exposures, predictors, potential | 4 |
|  |  | confounders, and effect modifiers. Give diagnostic criteria, if |  |
|  |  | applicable |  |
| Data sources/ | 8* | For each variable of interest, give sources of data and details | 4 |
| measurement |  | of methods of assessment (measurement). Describe |  |
|  |  | comparability of assessment methods if there is more than |  |
|  |  | one group |  |
| Bias | 9 | Describe any efforts to address potential sources of bias | 4 |
| Study size | 10 | Explain how the study size was arrived at | 4 |
| Quantitative variables | 11 | Explain how quantitative variables were handled in the | 4 |
|  |  | analyses. If applicable, describe which groupings were |  |
|  |  | chosen and why |  |
| Statistical methods | 12 | (*a*) Describe all statistical methods, including those used to | 5 |
|  |  | control for confounding |  |
|  |  | (*b*) Describe any methods used to examine subgroups and | 5 |
|  |  | interactions |  |
|  |  | (c) Explain how missing data were addressed | 5 |
|  |  | (*d*) If applicable, describe analytical methods taking account | Not applicable |
|  |  | of sampling strategy |  |
|  |  | (*e*) Describe any sensitivity analyses | Not applicable |
| **Results** |  |  |  |
| Participants | 13* | (a) Report numbers of individuals at each stage of study—eg | 5 |
|  |  | numbers potentially eligible, examined for eligibility, |  |
|  |  | confirmed eligible, included in the study, completing follow- |  |
|  |  | up, and analysed |  |
|  |  | (b) Give reasons for non-participation at each stage | 5 |
|  |  | (c) Consider use of a flow diagram | - |
| Descriptive data | 14* | (a) Give characteristics of study participants (eg | 5-8 |
|  |  | demographic, clinical, social) and information on exposures |  |
|  |  | and potential confounders |  |

|  |  | (b) Indicate number of participants with missing data for  each variable of interest | 5-8 |
| --- | --- | --- | --- |
| Outcome data | 15* | Report numbers of outcome events or summary measures | 5-8 |
| Main results | 16 | (*a*) Give unadjusted estimates and, if applicable, confounder- adjusted estimates and their precision (eg, 95% confidence  interval). Make clear which confounders were adjusted for and why they were included | 5-8 |
|  |  | (*b*) Report category boundaries when continuous variables were categorized | Not applicable |
|  |  | © If relevant, consider translating estimates of relative risk  into absolute risk for a meaningful time period | Not applicable |
| Other analyses | 17 | Report other analyses done—eg analyses of subgroups and  interactions, and sensitivity analyses | Not applicable |
| **Discussion** |  |  |  |
| Key results | 18 | Summarise key results with reference to study objectives | 9 |
| Limitations | 19 | Discuss limitations of the study, taking into account sources of potential bias or imprecision. Discuss both direction and  magnitude of any potential bias | 10-11 |
| Interpretation | 20 | Give a cautious overall interpretation of results considering objectives, limitations, multiplicity of analyses, results from  similar studies, and other relevant evidence | 10-11 |
| Generalisability | 21 | Discuss the generalisability (external validity) of the study  results | 10-11 |
| **Other information** |  |  |  |
| Funding | 22 | Give the source of funding and the role of the funders for the present study and, if applicable, for the original study on  which the present article is based | 12 |
